# *TRIM67* inhibits tumor proliferation and metastasis by mediating *MAPK11* in Colorectal Cancer

**DOI:** 10.7150/jca.47538

**Published:** 2020-08-18

**Authors:** Ying Liu, Guiqi Wang, Xia Jiang, Wei Li, Congjie Zhai, Fangjian Shang, Shihao Chen, Zengren Zhao, Weifang Yu

**Affiliations:** 1 Department of General Surgery, Hebei Key Laboratory of Colorectal Cancer Precision Diagnosis and Treatment, The First Hospital of Hebei Medical University, Donggang Road No.89, Shijiazhuang, Hebei 050031, P.R. China.; 2 Department of Endoscopy Center, The First Hospital of Hebei Medical University, Donggang Road No.89, Shijiazhuang, Hebei 050031, P.R. China.

**Keywords:** *TRIM67*, colorectal cancer, *MAPK11*, cell proliferation, metastasis

## Abstract

**Purpose:** We recently reported that tripartite motif-containing 67 (*TRIM67*) activates p53 to suppress colorectal cancer (CRC). However, the function and mechanism of *TRIM67* in the inhibition of CRC cell proliferation and metastasis remains to be further elucidated.

**Methods:** We detected the expression of *TRIM67* in CRC tissues compared with normal tissues and confirmed its relationship with clinicopathological features. DNA methylation of *TRIM67* was analyzed to determine its significantly hypermethylated sites in CRC tissues. CCK-8, colony formation, transwell migration, and Matrigel invasion assays were performed to evaluate the effects of *TRIM67* on cell proliferation and metastasis in CRC cells. RNA sequencing of *TRIM67* and *TRIM67* rescue experiments were performed to reveal its mechanisms in CRC cell proliferation and metastasis.

**Results:** *TRIM67* expression was significantly downregulated in CRC tissues and its expression was associated with clinical stage, invasive depth, tumor size, lymph node metastasis, and Dukes' stage. Three methylation sites were significantly hypermethylated and negatively correlated with *TRIM67* expression in CRC tissues. *TRIM67* suppressed proliferation, migration, and invasion in CRC cells. RNA sequencing revealed that protein mitogen-activated protein kinase 11 (*MAPK11*) was a potential downstream negative regulatory gene of *TRIM67*. Reversing *MAPK11* expression could rescue the effects of *TRIM67* on the proliferation and metastasis of CRC cells.

**Conclusion:** *TRIM67* inhibited cell proliferation and metastasis by mediating *MAPK11* in CRC, and may be a potential target to inhibit CRC metastasis.

## Introduction

Colorectal cancer (CRC) is a main cause of death worldwide, and its morbidity and mortality have increased in recent years 1. Multiple genes and signaling pathways have been identified but the precise molecular mechanism of CRC is not fully understood. Therefore, clarifying its pathogenesis and identifying novel molecular biomarkers to improve the diagnosis and prognosis of patients with CRC are urgently required 2.

The tripartite motif (TRIM) family is a large class of RBCC proteins of which nearly 70 members have been identified in humans, while fewer than 20 members have been identified in invertebrates and none in single-celled organisms. The high number of TRIM members in humans indicates that it is a rapidly evolving family 3. Members of the TRIM protein family are involved in many biological processes including proliferation, differentiation, development, autophagy, and apoptosis of cancer cells 4-8. In recent years, various studies have associated several TRIM family members, *TRIM15*, *TRIM27* and *TRIM29*, with CRC through AKT or JAK2/STAT3 signaling to regulate CRC cell migration and epithelial-mesenchymal transition 9-11.

*TRIM67*, a TRIM family member, is a protein-coding gene located at 1q42.2 consisting of approximately 59 kb DNA bases that encodes *TRIM67* (84 kDa) 12. To our knowledge, *TRIM67* has been reported to be involved in neuroprotective effects and tumor processes. Nicholas P. Boyer et al. 13 found that *TRIM67* is necessary for appropriate brain development and behavior. *TRIM67* is also associated with ubiquitination and interacts with *PRG-1* and *80K-H* to regulate the RAS signaling pathway to inhibit cell proliferation and enhance synapse production in neuroblastoma 14. Le Duy Do et al. 15 found that autoantibodies against *TRIM67* appeared to be specific biomarkers of paraneoplastic cerebellar degeneration and associated with lung carcinoma. A recent study implicated that *TRIM67* activates the NF-κB pathway and promotes cell apoptosis in GA-13315-treated lung cancer cells 16. Our previous study found that in The Cancer Genome Atlas (TCGA)*, TRIM67* is frequently methylated in CRC compared with adjacent normal tissues, and prevents intestinal tumorigenesis in mice and improves chemotherapy efficacy by activating the *TRIM67*-p53 axis 17. In the present study, *TRIM67* expression levels and clinicopathological features based on TCGA dataset and our cohort was further examined. We also investigated the direct functions of *TRIM67* in CRC cells and studied its potential mechanisms as a tumor suppressor in CRC.

## Materials and Methods

### TCGA data download and analysis

mRNA sequencing data of 647 CRC tissues and 51 normal tissues was downloaded from TCGA (https://tcga-data.nci.nih.gov/), and data for the mRNA expression of 19,640 genes were obtained. Immunohistochemistry (IHC) data based on TCGA was downloaded from The Human Protein Atlas (https://www.proteinatlas.org/). The staining intensity was scored as negative (0), weak (1), moderate (2), or strong (3). The staining quantity was scored as 1 (≤10%), 2 (11%-50%), 3 (51%-75%), or 4 (>75%). The total immunostaining score was calculated by multiplying the staining intensity score with the quantity and ranged from 0 to 12 18.

### Patients and tissue specimens

The present study was approved by the Ethics Committee of The First Hospital of Hebei Medical University (No. 2016004) and was in accordance with the principles of Declaration of Helsinki. A total of 60 pairs of human CRC tissues and adjacent normal tissues were collected from the First Hospital of Hebei Medical University (Shijiazhuang, Hebei, China). All patients were without any therapeutic invention and had signed informed consent prior to surgery. All cancer tissues were histologically confirmed as colorectal adenocarcinoma or mucinous carcinoma and were immediately preserved in liquid nitrogen within 5 min following resection and then placed at -80°C for long-term preservation.

### DNA methylation assay

Genomic DNA (gDNA) was extracted from 10 pairs of human CRC tissues and adjacent normal tissues using the DNeasy Blood and Tissue Kit (Qiagen, Valencia, CA, USA) according to the manufacturer's protocol. DNA quality assessment and quantification were performed using the NanoDrop 1000 (Thermo Scientific). Bisulphite conversion was performed according to the manufacturer's recommendations for the Illumina Infinium Assay (EZ DNA methylation kit, ZYMO, Orange, CA). The converted and amplified gDNA fragment was used for hybridization with the 15-mers specific capture probe (Table 2) according to the Illumina Infinium HD methylation protocol for genomic facilities (Novogene, Beijing, China). The nucleotide substrates (A/T and C/G) were labeled by different fluorescent dyes and only the probe with complementary binding of gDNA could be single base extended. Finally, iScan software was used to read and output the methylation level results according to the fluorescence.

### Cell culture and transfection

Human CRC cell lines HCT116, SW480, LoVo, SW620, SW1116, Caco2, SW1463, and HT29 cell lines were obtained from Prof. Jun Yu (The Chinese University of Hong Kong, Hong Kong, China). HCT116 and HT29 were cultured in McCoy's 5A medium (Gibco, Grand Island, NY, USA) and the other cells lines were cultured in DMEM medium (Gibco), supplemented with 10% fetal bovine serum (FBS; Gibco) and 1% penicillin-streptomycin (Invitrogen, Carlsbad, CA, USA) in a 37°C humidified incubator with 95% air and 5% CO_2_.

PLV-*TRIM67*-shRNA and negative control plasmids were obtained from Prof. Jun Yu. M98-*TRIM67*, M98-*MAPK11*, si*MAPK11*, and negative control plasmids were purchased from GeneCopoeia (Guangzhou, China). The plasmids were transfected into CRC cells using Lipofectamine 2000 (Invitrogen) according to the manufacturer's protocol.

### Reverse transcription-quantitative polymerase chain reaction (RT-qPCR analysis)

Total RNA was extracted from frozen tissues and cells using TRIzol^®^ reagent (Invitrogen) according to the manufacturer's protocol. cDNA was produced from the RNA by performing reverse transcription using a PrimeScript™ RT Reagent Kit (Perfect Real Time; Takara, Otsu, Japan). The RT-qPCR experiment was performed using AceQ qPCR SYBR Green Master Mix (Vazyme, Nanjing, China) according to the manufacturer's protocol. The final reaction was performed with a 7500 Real-Time PCR system (Applied Biosystems, Foster City, CA, USA) using the following protocol: hot-start DNA polymerase activation to 95°C for 5 min; 40 cycles of 95°C for 10 sec, and 60°C for 30 sec. The specific primers were as follows: *TRIM67*, forward, 5'-TCCCAACTGTTTGCCACAGG-3' and reverse, 5'-AGGTTAGAACGGAACGCCTC-3'; *MAPK11*, forward, 5'-CGACGAGCACGTTCAATTCC-3' and reverse, 5'-TCACAGTCCTCGTTCACAGC-3'; *GAPDH*, forward, 5'-AGCCTCAAGATCATCAGCAATG-3' and reverse, 5'-TGTGGTCATGAGTCCTTCCACG-3'. The expression of *TRIM67* and *MAPK11* was normalized to *GAPDH* and relative expression levels were calculated using the ^2-∆∆^Ct method.

### Western blot

Forty-eight hours after transfection, total cellular proteins were extracted using sodium dodecyl sulfate sample buffer and lysates were processed for western blot analysis. Equal amounts of total protein from each group were separated via SDS-PAGE and transferred to polyvinylidene difluoride membranes (PVDF, Millipore, Darmstadt, Germany), which were blocked with 5% skimmed milk in PBS-T for 2 h. The membranes were incubated with primary antibodies specific to *TRIM67* (1:200; Abcam, Cambrige, MA, USA), *MAPK11* (1:1000; Abcam), and β-actin (1:3500; Proteintech, Wuhan, China) at 4°C overnight. The membranes were then incubated with secondary DyLight fluorescent dye-conjugated antibodies (1:2500; Invitrogen) for 1 h at room temperature. Protein signals were detected and scanned by the Odyssey CLx Imaging System (LI-COR Biosciences, Lincoln, USA).

### Cell proliferation and colony formation assay

For the proliferation assay, transfected cells were seeded into 96-well plates and cultured for 24 h. Then 10 μl cell counting kit-8 (CCK-8) reagent (Dojindo, Kumamoto, Japan) was added to each well according to the manufacturer's protocol 24 h. Quadruplicate OD450 values were determined by Promega GloMax Luminescence detector after 2 h incubation. For the colony formation assay, transfected cells were seeded into 10-cm plates. The visible colonies were fixed with 4% formaldehyde and stained with crystal violet after 12 days. Colonies containing more than 50 cells were counted and each condition was performed in triplicate.

### Migration and invasion assays

Cell migration and invasion assays were performed using 8 µm pores (BD Biosciences, Franklin Lakes, NJ, USA). Briefly, 2×10^5^ cells were resuspended with serum-free medium in the upper chambers of transwell plates coated with or without Matrigel (354480; BD), and medium containing 10% FBS was added to the bottom chamber. After 24 h, cells that had successfully translocated across the membrane were fixed and stained with Diff-Quick stain kit, and counted in three randomly selected fields under a light microscope.

### RNA sequencing

Total RNA from transfected cells was quantified by a NanoDrop 1000 (Thermo Scientific, Wilmington, DE, USA). The quality and integrity of the total RNA were assessed by an Agilent 2100 Bioanalyzer (Agilent Technologies, Santa Clara, CA, USA). The Illumina Xten platform was used for high-throughput next-generation sequencing by Sangon Biotech Co., Ltd (Shanghai, China). Differentially expressed genes (DEGs) were identified as such if the log_2_ fold change >1 or < -1 and *P*<0.05. Gene Ontology (GO) enrichment and enriched Kyoto Encyclopedia of Genes and Genomes (KEGG) pathway analyses were performed.

### Statistical analysis

The results of normal distribution data are expressed as mean ± SD and the results of non-normal distribution data are expressed as median and quartile spacing. Student's *t*-test, one-way ANOVA, two-way ANOVA, and Pearson correlation analysis were used in this study. All statistical analyses were performed using GraphPad Prism 8 (GraphPad Software, USA), SPSS Statistics 21 (IBM, NY, USA). *P*<0.05 was considered as statistically significant.

## Results

### *TRIM67* expression was downregulated and was associated with clinical stage, invasive depth, tumor size, lymph node metastasis, and Dukes' stage in CRC patients

**Figure 1:**
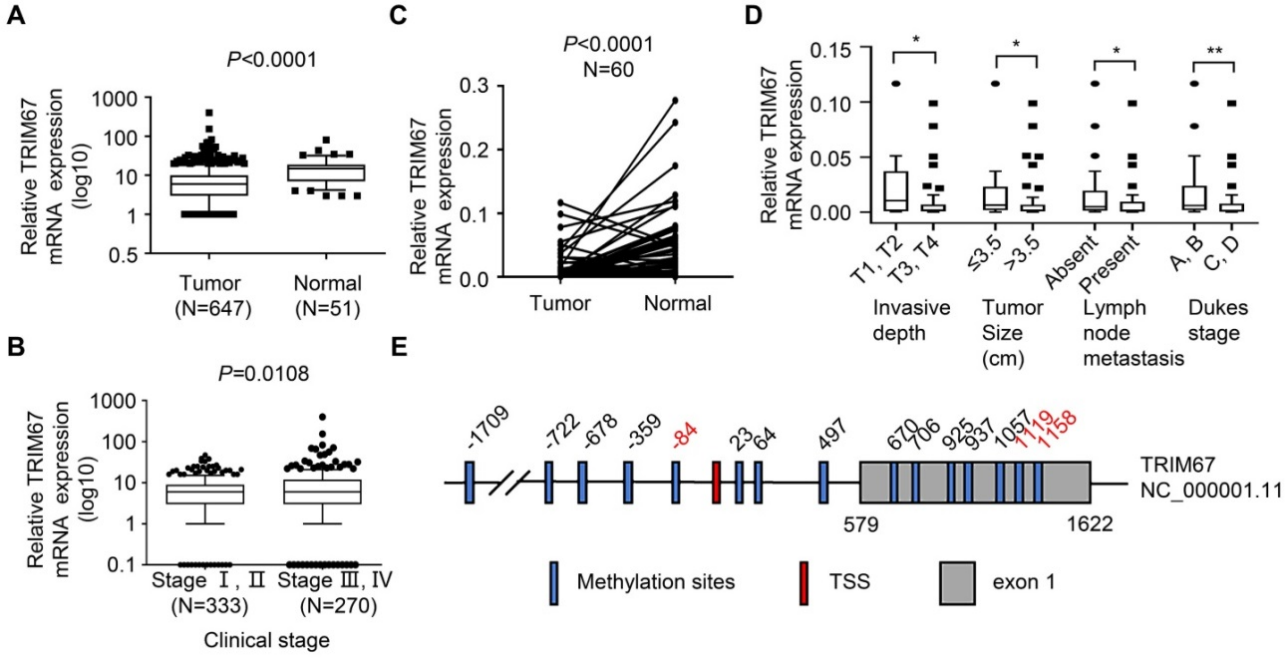
***TRIM67* expression is downregulated and is significantly associated with clinical stage, invasive depth, tumor size, lymph node metastasis, and Dukes' stage in human CRC tissues. A** *TRIM67* expression in CRC (n=647) and normal tissues (n=51) in TCGA cohort. **B** Relationship between *TRIM67* expression and clinical stage in CRC based on TCGA cohort. **C** *TRIM67* expression in paired CRC and normal tissues (n=60) from our cohort. **D** Relationship between *TRIM67* expression and invasive depth, tumor size, lymph node metastasis, and Dukes' stage in CRC based on our cohort. **E** Schematic presentation of *TRIM67* DNA methylation sites; significantly different methylation sites (FC>1.5, *P*<0.05) are shown in red. **P<0.01, *P<0.05.

To examine *TRIM67* expression levels in CRC, we first analyzed* TRIM67* mRNA expression in 647 CRC tissues and 51 normal tissues from TCGA datasets. *TRIM67* expression was significantly downregulated in CRC tissues compared with normal tissues. (*P*<0.0001; Fig. 1A). Next, we investigated whether *TRIM67* expression was associated with the clinical stage of CRC patients. As shown in Figure 1B, *TRIM67* expression levels of clinical stage I-II (n=333) was significantly higher than stage III-IV (n=270) CRC patients *(P*=0.0108) in TCGA cohort. Then we evaluated 60 pairs of CRC tissues and matched normal tissues in our cohort using RT-qPCR analysis. The results were consistent with TCGA cohort findings in that *TRIM67* mRNA levels were significantly downregulated in CRC tissues than in normal tissues (*P*<0.0001; Fig. 1C). Further, the clinicopathological characteristics analysis of our cohort showed that *TRIM67* expression was significantly correlated with invasive depth (*P*=0.0456), tumor size (*P*=0.0432), lymph node metastasis (*P*=0.0330), and Dukes' stage (*P*=0.0074; Fig. 1D), but not with sex, age, tumor location, distant metastasis, pathology, and differentiation grade (*P*>0.05; Table 1). In summary, higher *TRIM67* expression was more likely to be detected in normal tissues, as well as earlier clinical stage, less invasive depth (T1, T2), smaller tumor size (≤3.5 cm), absent lymph node metastasis, and Dukes' stage A-B CRC tissues. These results indicated that *TRIM67* may have a tumor suppressor role and act as a potential prognostic marker in CRC.

To explore the mechanism of TRIM67 downregulation in CRC tissues, DNA methylation of the TRIM67 gene was analyzed. Fifteen DNA methylation sites are included around the transcription start site (TSS) (±2000 bp) of the TRIM67 gene (Fig. 1E). DNA hypermethylation was identified in three DNA methylation sites, and was significantly negative correlated with TRIM67 expression (FC>1.5, P<0.05; Table 2).

### *TRIM67* suppressed the proliferation, colony formation ability, migration, and invasion in CRC cells

**Figure 2:**
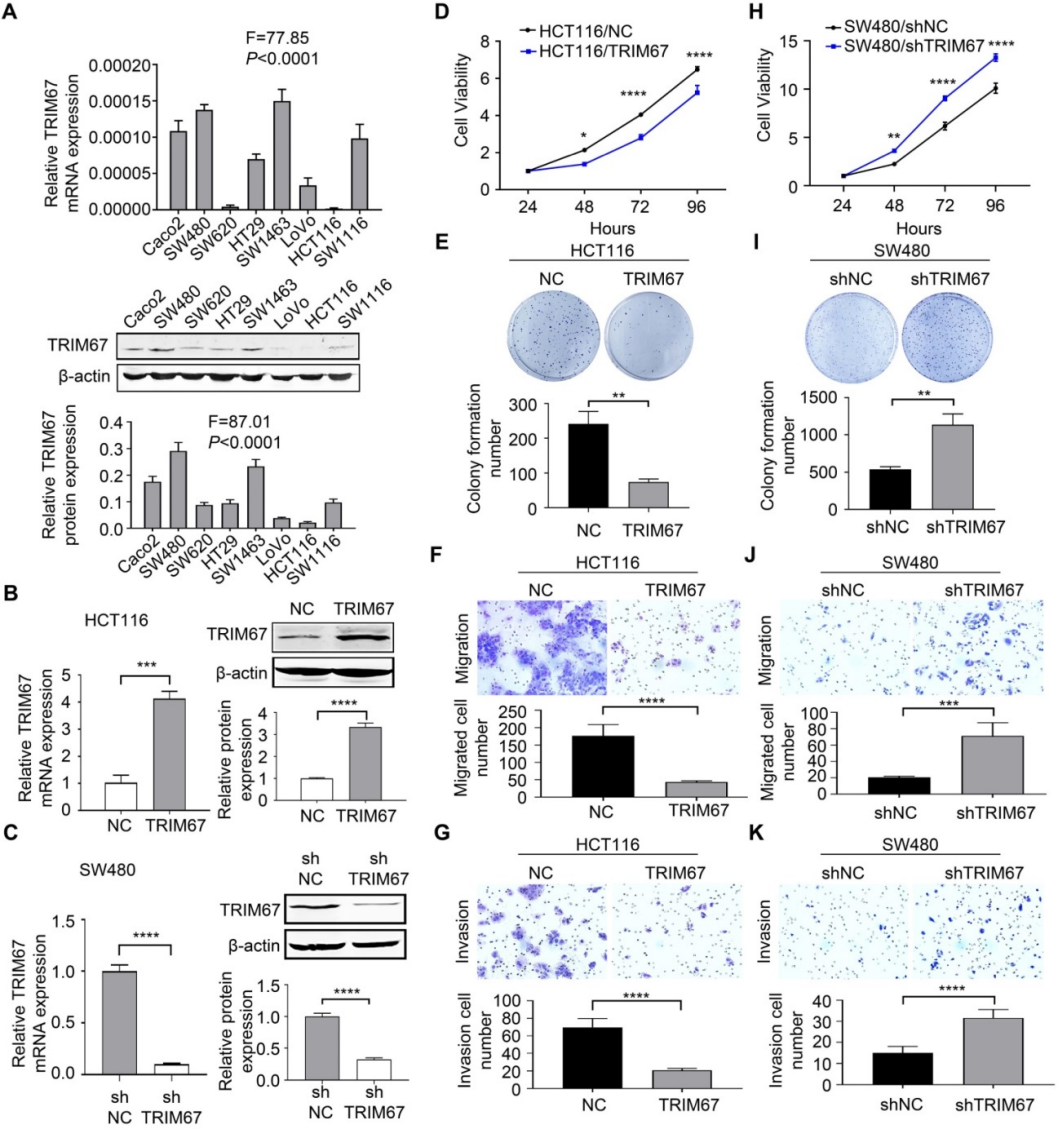
***TRIM67* suppressed cell proliferation, colony formation, migration, and invasion in CRC cells. A** *TRIM67* expression in eight CRC cell lines was detected by RT-qPCR and western blot. **B** *TRIM67* overexpression in HCT116 cells was detected by RT-qPCR and western blot. **C** *TRIM67* knockdown in SW480 cells expression was detected by RT-qPCR and western blot. **D** Effects of *TRIM67* overexpression on cell viability in HCT116 cells detected by CCK-8 assay. **E** Effects of *TRIM67* overexpression on HCT116 colony formation assays. **F** Effects of *TRIM67* overexpression on HCT116 cell migration assays. **G** Effects of *TRIM67* overexpression on HCT116 cell invasion assays. **H** Effects of *TRIM67* silencing on SW480 cell viability detected by CCK-8 assay. **I** Effects of *TRIM67* silencing on SW480 cell colony formation assays. **J** Effects of *TRIM67* silencing on SW480 cell migration. **K** Effects of *TRIM67* silencing on SW480 cell invasion assays. Results show mean ± SD from three independent experiments. ****P<0.0001, ***P<0.001, **P<0.01, *P<0.05.

To explore the role of *TRIM67* in the biological behavior of CRC, we examined eight human CRC cell lines for *TRIM67* expression. The results showed that *TRIM67* was expressed at low levels in HCT116 cells and was highly expressed in SW480 cells (*P*<0.0001; Fig. 2A). Thus, *TRIM67* was overexpressed in HCT116 cells using M98-*TRIM67* plasmid whereas it was knocked down in SW480 using PLV-*TRIM67*-shRNA plasmid. Cells of the control group were transduced with negative control plasmid. Altered *TRIM67* expression was examined by qRT-PCR and western blot (*P*<0.001; Fig. 2B and 2C).

Cell viability, colony formation ability, migration, and invasion was analyzed by CCK-8 assay, colony formation assay, transwell migration, and Matrigel invasion assays, respectively. These revealed that *TRIM67* overexpression significantly decreased the viability (*P*<0.0001; Fig. 2D), colony formation (*P*<0.01; Fig. 2E), migration (*P*<0.0001; Fig. 2F), and invasion abilities (*P*<0.0001; Fig. 2G) of HCT116 cells compared with control cells. Conversely, *TRIM67* knockdown significantly increased the viability (*P*<0.0001; Fig. 2H), colony formation (*P*<0.01; Fig. 2I), migration (*P*<0.001; Fig. 2J), and invasion abilities (*P*<0.0001; Fig. 2K) of SW480 cells compared with control cells. Therefore, we concluded that *TRIM67* inhibits cell viability, colony formation ability, migration, and invasion, and has a tumor suppressor role in CRC cells.

### *MAPK11* is a potential downstream negative regulatory gene of *TRIM67* in CRC

**Figure 3:**
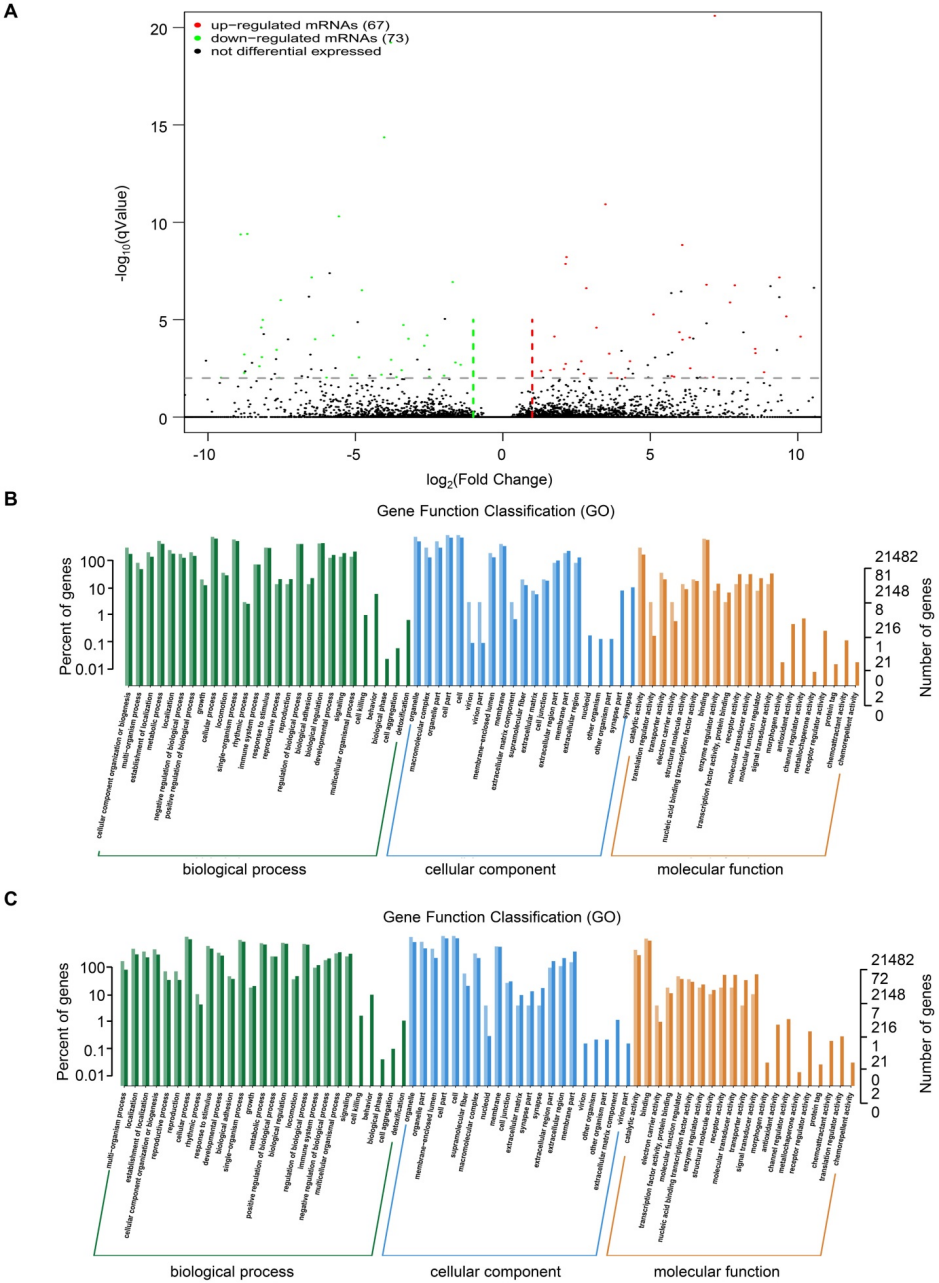
** GO analysis of DEGs from RNA sequencing data of *TRIM67* in CRC. A** Volcano plot of upregulated (red spots, n=67) and downregulated mRNAs (green spots, n=73) from RNA sequencing data of *TRIM67*-overexpressing and control HCT116 cells. **B** GO analysis based on DEGs upregulated by *TRIM67* from RNA sequencing data of *TRIM67*-overexpressing and control HCT116 cells. **C** GO analysis based on DEGs downregulated by *TRIM67* from RNA sequencing data of *TRIM67*-overexpressing and control HCT116 cells.

To investigate the mechanism of *TRIM67* as a tumor suppressor in CRC cells, we performed RNA sequencing in HCT116 cells overexpressing *TRIM67* or control vector. A total of 140 DEGs were identified, of which 67 were upregulated and 73 were downregulated (*P*<0.05; Fig. 3A). Detailed information of the top 20 upregulated mRNAs (log_2_ fold change>1, *P*<0.05) and top 20 downregulated mRNAs (log_2_ fold change< -1, *P*<0.05) are shown in Table 3 and 4.

**Fig 4:**
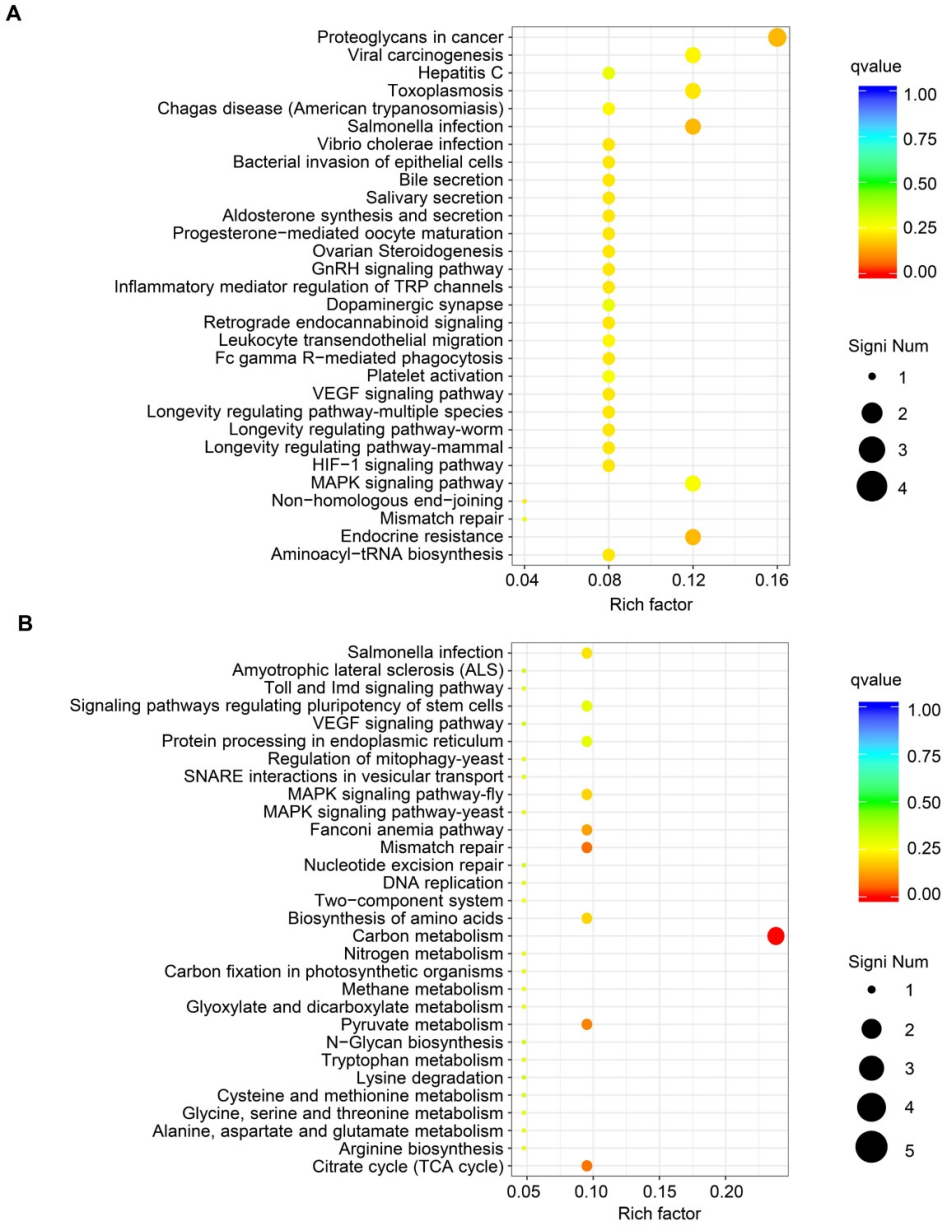
**KEGG pathway analysis of DEGs from RNA sequencing data of *TRIM67* in CRC. A** KEGG pathway analysis based on DEGs upregulated by *TRIM67* from RNA sequencing data of *TRIM67*-overexpressing and control HCT116 cells.** B** KEGG pathway analysis based on DEGs downregulated by *TRIM67* from RNA sequencing data of *TRIM67*-overexpressing and control HCT116 cells.

GO functional analysis of DEGs showed that the biological processes they were significantly enriched in were cellular processes, metabolic processes, developmental processes, signaling, growth, and biological adhesion. The cellular components they were most located in were cell, organelle, membrane, extracellular region, cell junction. Furthermore, the molecular functions they were involved in were binding, molecular function regulation, transporter activity, and receptor activity. (Fig. 3B and Fig. 3C). KEGG pathway analysis of upregulated mRNAs revealed that they were significantly enriched in proteoglycans in cancer and its significant symbols *PRKACB*, *PXN*, *RPS6KB2*, and *MAPK11*, VEGF signaling pathway and its significant symbols *PXN* and *MAPK11*, and *Salmonella* infection and its significant symbols *KLC1*, *IFNGR2*, and *MAPK11* (Fig. 4A). Downregulated mRNAs were significantly enriched in carbon metabolism and its significant symbols *MDH2*, *OGDH*, *PSPH*, *ME2*, and *CPS1*, mismatch repair and its significant symbols *RFC3* and *PMS2*, and MAPK signaling pathway and its significant symbols *TBL1X* and *MAPK11* (Fig. 4B). Above all, *MAPK11* (log_2_ fold change=-16.55, *P*<0.05; Table 4) was revealed to be one of the most significant downstream target genes regulated by *TRIM67*.

**Figure 5:**
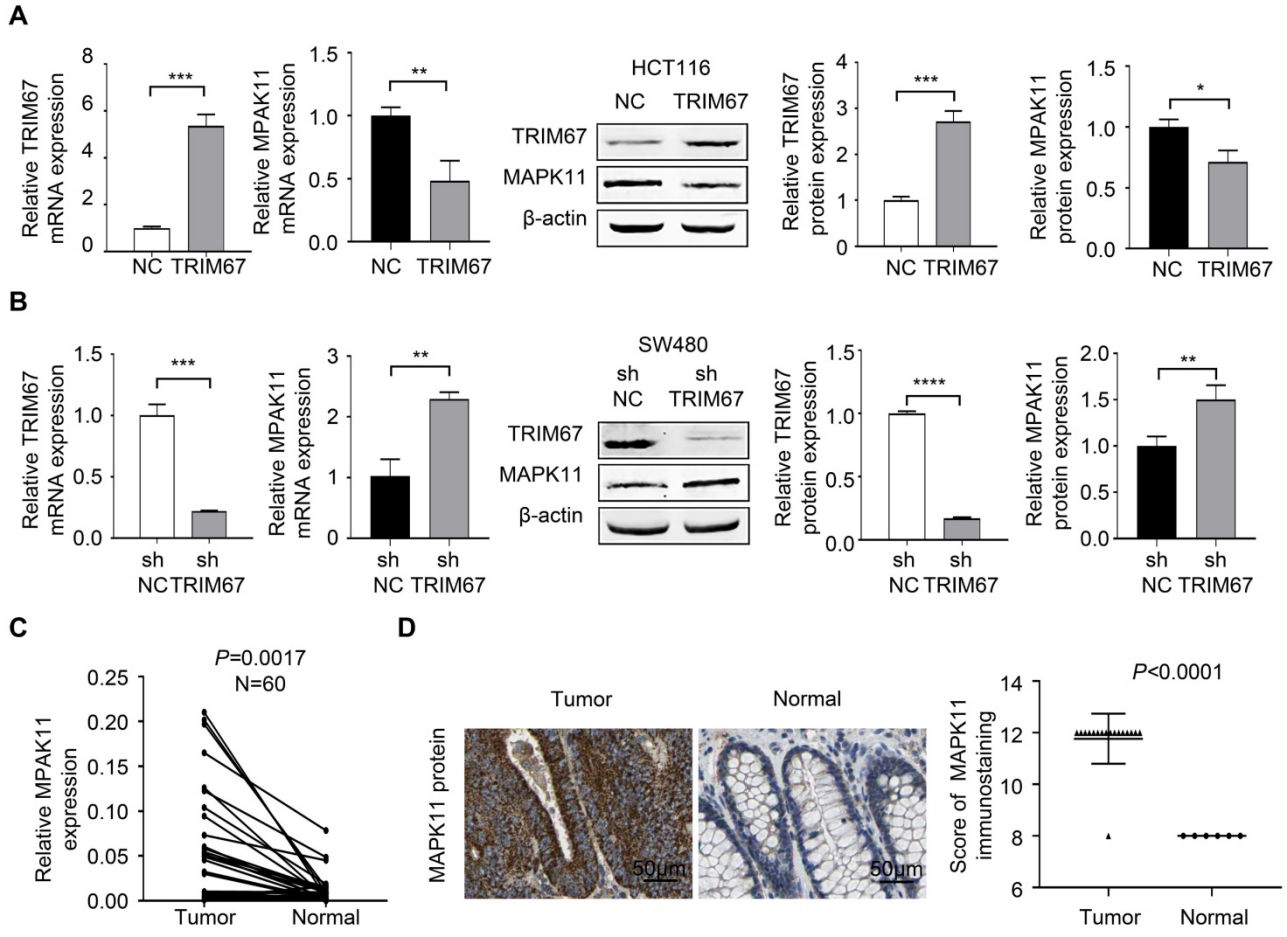
***TRIM67* negatively regulated *MAPK11* expression in CRC. A** *MAPK11* expression was detected by RT-qPCR and western blot in *TRIM67*-overexpressing HCT116 cells. **B** *MAPK11* expression was detected by RT-qPCR and western blot in *TRIM67*-silenced SW480 cells. **C*** MAPK11* expression in paired CRC and normal tissues (n=60) from our cohort was detected by RT-qPCR. **D** *MAPK11* expression in CRC and normal tissues from TCGA cohort was detected by IHC. Results show mean ± SD from three independent experiments. ****P<0.0001, ***P<0.001, **P<0.01, *P<0.05.

To investigate *MAPK11* was a potential downstream negative regulatory gene of *TRIM67*, we performed the experiment of *TRIM67* overexpression using M98-*TRIM67* plasmid in HCT116 cells, and performed *TRIM67* knockdown using PLV-*TRIM67*-shRNA plasmid in SW480 cells. RT-qPCR and western blot were used to analyze the regulation of *MAPK11* by altering *TRIM67* expression. As shown in Figure 5A, *MAPK11* expression was downregulated in *TRIM67*-overexpressed HCT116 cells (*P*<0.05) while upregulated in *TRIM67*-silenced SW480 cells (*P*<0.01; Fig. 5B). Moreover, in our cohort, the expression of *MAPK11* in cancer tissues was higher than in paired normal tissues (*P*=0.0017; Fig. 5C). Similarly, in TCGA cohort, *MAPK11* expression was higher than in paired normal tissues (*P*<0.0001); representative images of *MAPK11* staining are shown in Figure 5D.

### Reversing *MAPK11* expression can rescue the effects of *TRIM67* on CRC cell proliferation, colony formation ability, migration, and invasion

**Figure 6:**
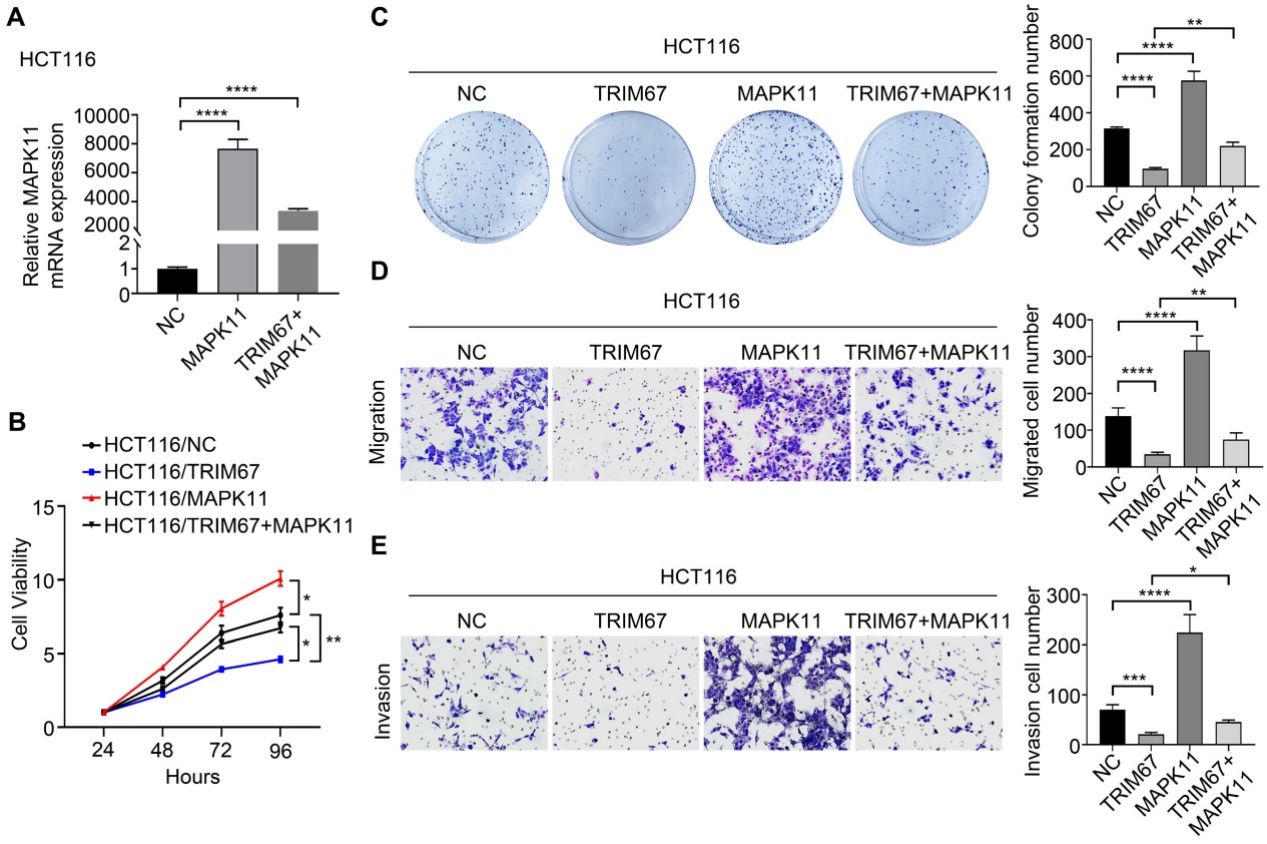
***MAPK11* reversed the influence of *TRIM67* on cell proliferation, colony formation, migration, and invasion in HCT116 cells. A** Co-regulation of *TRIM67* and/or* MAPK11* was detected by RT-qPCR. **B** Effects of co-regulation of *TRIM67* and/or *MAPK11* on HCT116 cell viability were detected by CCK-8 assay. **C** Effects of co-regulation of *TRIM67* and/or *MAPK11* on HCT116 cell colony formation assays. **D** Effects of co-regulation of *TRIM67* and/or *MAPK11* on HCT116 cell migration assays. **E** Effects of co-regulation of *TRIM67* and/or *MAPK11* on HCT116 cell invasion assays. Results show mean ± SD from three independent experiments. ****P<0.0001, ***P<0.001, **P<0.01, *P<0.05.

**Figure 7:**
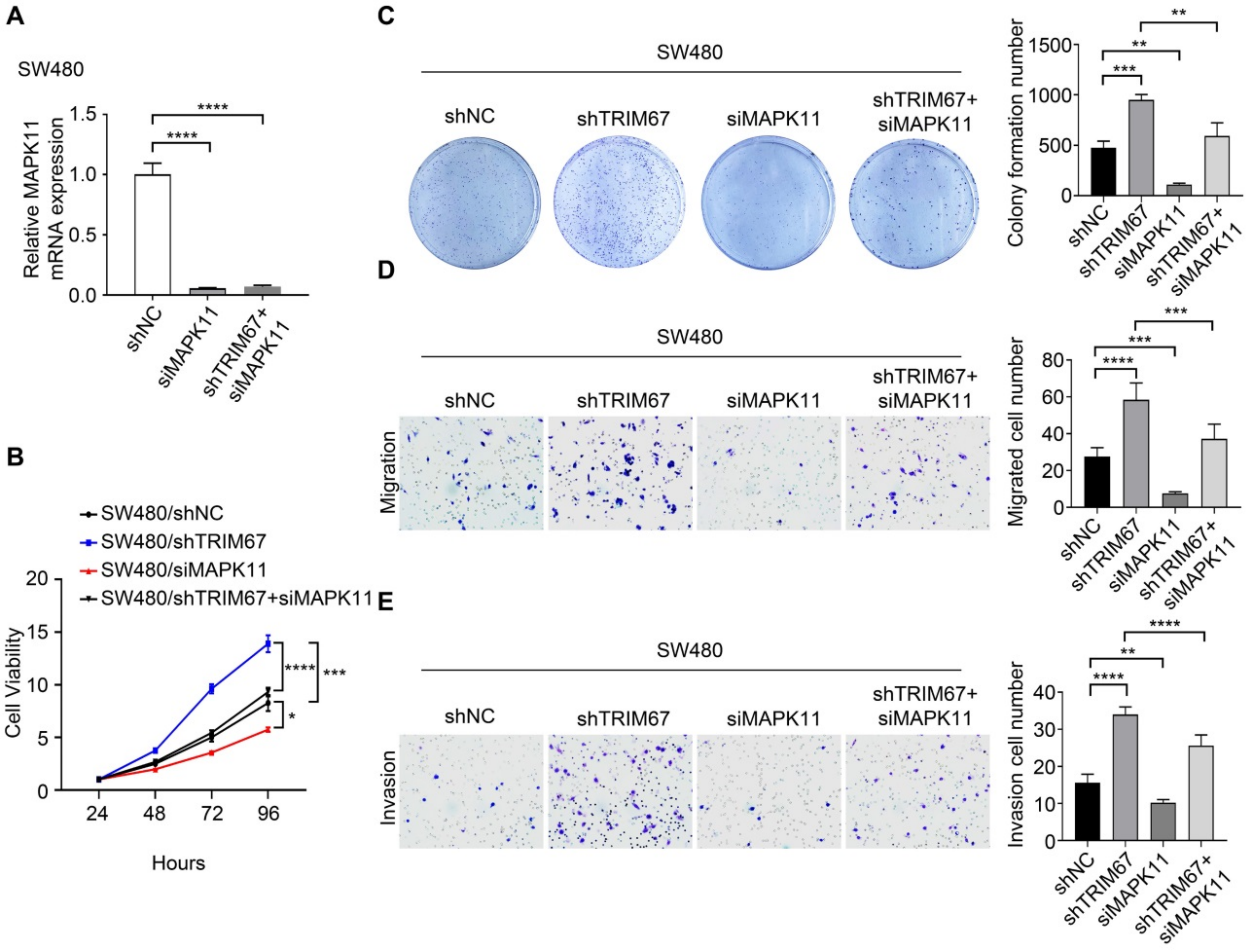
***MAPK11* reversed the effect of *TRIM67* on cell proliferation, colony formation, migration, and invasion in SW480 cells. *TRIM67* and *MAPK11* were knocked down in SW480 cells. A** Co-depletion of *TRIM67* and/or *MAPK11* was detected by RT-qPCR. **B** Effects on SW480 cell viability were detected by CCK-8 assay. **C** Effects on SW480 colony formation were detected by colony formation assay. **D** Effects on SW480 cell migration were detected by cell migration assay. **E** Effects on SW480 cell invasion were detected by invasion assay. Results show mean ± SD from three independent experiments. ****P<0.0001, ***P<0.001, **P<0.01, *P<0.0.

Co-overexpression of both *TRIM67* and *MAPK11* in HCT116 cells and co-depletion of both *TRIM67* and *MAPK11* in SW480 cells was performed to investigate whether *MAPK11* mediates the influence of *TRIM67* on the biological behavior of CRC cells. Altered *MAPK11* expression was examined by qRT-PCR (*P*<0.0001; Fig. 6A). The results showed that overexpression of *TRIM67* significantly decreased the proliferation (*P*<0.01; Fig. 6B), colony formation ability (*P*<0.0001; Fig. 6C), migration (*P*<0.0001; Fig. 6D), and invasion (*P*<0.001, Fig. 6E) of HCT116 cells, while overexpression of *MAPK11* significantly increased the proliferation, colony formation ability, migration, and invasion of HCT116 cells (*P*<0.05; Fig. 6A-6E). Compared with overexpression of *TRIM67* alone, co-overexpression of both *TRIM67* and *MAPK11* restored cell proliferation, colony formation ability, migration, and invasion to levels near to control cells (*P*<0.05; Fig. 6A-E). The depletion of *TRIM67* and/or *MAPK11* resulted in the opposite effects in SW480 cells (*P*<0.05; Fig. 7A-E). These results revealed that reversing *MAPK11* expression could rescue the inhibitory effects of *TRIM67* on cell proliferation, colony formation ability, migration, and invasion in CRC.

## Discussion

In this study, further examination of *TRIM67* expression demonstrated that it was downregulated in CRC tissues compared with matched normal tissues in our cohort. Furthermore, higher *TRIM67* expression was associated with earlier clinical stage and Dukes' stage, less invasive depth and lymph node metastasis, smaller tumor size. To our knowledge, this is the first study to reveal *TRIM67* expression and clinicopathological features in CRC, indicating that *TRIM67* may have a tumor suppressor role as well as acting as a prognostic biomarker for CRC.

Previous studies and the present study demonstrated that *TRIM67* suppresses colorectal carcinogenesis and tumor growth *in vivo*, and plays tumor suppressor role in multiple biological functions including proliferation, colony formation ability, apoptosis, cell cycle, migration, and invasion *in vitro*. Mechanically, the previous study found that *TRIM67* activates the p53 signaling pathway to suppress CRC initiation and progression. To gain insights into mechanism of *TRIM67* as a CRC suppressor, RNA sequencing in present study revealed that *TRIM67* targeted *MAPK11* to play a tumor suppressor role in CRC cells. Studies have showed that MAPK signaling pathways are involved in tumor progression, cell growth, cell proliferation, and metastasis in CRC 19-22. The MAPK pathways include extracellular-regulated kinases 1 and 2 (*ERK1*/*ERK2*), c-Jun N-terminal kinases (*JNKs*), p38 (*MAPK11*-*14*), and extracellular-regulated kinase 5 (*ERK5*). p38 comprises p38β (*MAPK11*), p38γ (*MAPK12*), p38δ (*MAPK13*), and p38α (*MAPK14*), which are involved in numerous cellular functions including proliferation, apoptosis, cell cycle, autophagy, and metastases 23, 24. *MAPK11* was shown to be upregulated in hepatocellular carcinoma 25 and is a target gene of *miR-516a-5p* sponged by *circ-0001955* to facilitate hepatocellular carcinoma tumorigenesis 26. *MAPK11* is also highly expressed in breast cancer cells and enhances osteoclastogenesis and bone resorption 27. In lung cancer, *MAPK11* is involved in the p38 MAPK signaling pathway, and a p38-specific inhibitor decreases cell viability and enhances cisplatin sensitivity 28. These studies suggest that* MAPK11* is an oncogene that functions in diverse cancers. In the present study, we found that *MAPK11* was upregulated in CRC tissues compared with normal tissues, and that overexpression or depletion of *TRIM67* had a negative regulatory effect on the expression of *MAPK11* in CRC cell lines. Moreover, co-regulation of *TRIM67* and *MAPK11* expression reversed the impact of regulating *TRIM67* expression alone on the biological behavior of CRC cells, indicating that *MAPK11* could be a downstream negative regulatory gene of *TRIM67* and might be involved in *TRIM67*-mediated biological behavior in CRC cells. Studies have revealed that *TRIM45* inhibits the transcriptional activities of *ElK-1* and *AP-1* and acts as a negative transcriptional regulator in MAPK-mediated signaling 29 or functions as a member of the negative feedback loop of MAPK signaling 30. We speculated that *TRIM67* might be a negative transcriptional regulator in MAPK signaling and inhibits the phosphorylation of target transcription factors, negatively regulating the expression of *MAPK11* in CRC cells.

Furthermore, KEGG pathway analysis of the identified DEGs revealed that they are also enriched in other CRC-associated pathways. Proteoglycans in cancer and VEGF signaling were associated with *miR-181* target genes enriched in CRC with poor prognosis 31. Sophocarpine inhibited CRC cell migration via downregulation of the MEK/ERK/VEGF pathway 32, and pyrosoda curcumin exerted inhibition of angiogenesis through modulation of VEGF signaling regulatory miRNAs 33. Defective DNA mismatch repair was associated with genomic instability, which is one of the main mechanisms in CRC 34. It was reported that the downstream genes of the above CRC-related signaling pathways have important roles in CRC. *PXN* was revealed to be regulated by *miR-137* to promote tumor progression and metastasis in CRC 35. Another downstream gene, *PSPH*, was overexpressed in tumor tissues and was positively correlated with depth of invasion and distant metastasis in CRC 36. Moreover, *RFC3* mutation and loss occurred in large fractions of CRC, which contributed to cancer pathogenesis by deregulating DNA repair and replication 37. According to the evidence above, we speculate that the potential downstream regulatory genes of *TRIM67* in CRC might include *PXN*, *PSPH*, and *RFC3*, in addition to *MAPK11*, and might be involved in proteoglycans in cancer, VEGF, mismatch repair, or other signaling pathways to inhibit the proliferation and metastasis of CRC.

In conclusion, our data provide important insights into the tumor suppressive role of *TRIM67* in CRC. We demonstrated that *TRIM67* expression is correlated with clinical stage, invasive depth, tumor size, lymph node metastasis, and Dukes' stage in CRC. Moreover, *TRIM67* inhibits tumor proliferation, colony formation ability, migration, and invasion via regulation of *MAPK11*. In future, further studies are necessary to identify direct binding sites, transcription factors, or other molecular compounds that are involved in the interaction of *TRIM67* and *MAPK11*, as well as other potential downstream target genes and additional underlying mechanisms of *TRIM67*-mediated tumor suppressor functions. Besides, more analysis and clinical research are in need to clarify the functions of *TRIM67* as a potential biomarker and promising therapeutic target for CRC.

## Figures and Tables

**Table 1:** Relationship between *TRIM67* expression level and clinicopathological features in 60 CRC patients

Variables	Case (n=60)	*TRIM67* expression	*U*	*P*
**Sex**			289	0.1118
Male	41	0.0016 (0.0005, 0.0066)		
Female	19	0.0061 (0.0011, 0.0235)		
**Age**			353	0.2368
≤65	36	0.0028 (0.0006, 0.0150)		
>65	24	0.0014 (0.0005, 0.0086)		
**Tumor location**			430	0.7718
Colon	30	0.0025 (0.0005, 0.0109)		
Rectum	30	0.0022 (0.0004, 0.0132)		
**Invasive depth**			165	0.0456
T1, T2	11	0.0103 (0.0008, 0.0370)		
T3, T4	49	0.0017 (0.0005, 0.0069)		
**Tumor size (cm)**			242.5	0.0432
≤3.5	17	0.0063 (0.0017, 0.0231)		
>3.5	43	0.0015 (0.0005, 0.0068)		
**Lymph node metastasis**			304.5	0.0330
Absent	28	0.0049 (0.0012, 0.0194)		
Present	32	0.0014 (0.0003, 0.0095)		
**Distant metastasis**			253.5	0.7653
Absent	49	0.0023 (0.0005, 0.0098)		
Present	11	0.0026 (0.0004, 0.0155)		
**Dukes**			264	0.0074
A, B	26	0.0059 (0.0017, 0.0238)		
C, D	34	0.0013 (0.0003, 0.0077)		
**Pathology**			158	0.0686
Adenocarcinoma	50	0.0017 (0.0004, 0.0095)		
Mucinous carcinoma	10	0.0064 (0.0015, 0.0268)		
**Differentiation grade**			328.5	0.0827
Well	33	0.0034 (0.0010, 0.0145)		
Moderate, worse	27	0.0014 (0.0004, 0.0068)		

**Table 2:** Methylation sites of *TRIM67* in CRC tissues

No.	Site	Methylation		Pearson's correlation		ProbeSeq
		FC	*P*	*R*	*P*	
**1**	-1709	0.83	0.005	0.291	0.213	AATAAACTAAACCTAACAAAATTAAAATCTTATAAAAAAAACCCCTAACC
**2**	-722	1.37	0.045	-0.434	0.056	AATCCCCRAAATAAATAACACTTAAACTAACAAACAAAACAATAAAAAAC
**3**	-678	1.20	0.011	-0.500	0.025	AAAACATCACACTACTCACAAAACATAACTACTAACTCTAACAAAATACA (U) AAAACGTCGCACTACTCGCAAAACGTAACTACTAACTCTAACGAAATACG (M)
**4**	-359	1.31	0.028	-0.484	0.031	TTAAAAAACTTAAAACTCRAAATATAAAACCCRTAACCRCTATCTATCTC
**5**	**-84**	**1.64**	**0.009**	**-0.543**	**0.013**	AACRCAAAACAAACAAACATTATAAAAACAAAACAAAATAATAAACTCCC
**6**	23	1.46	0.013	-0.433	0.056	CAACACTACTCTACACTAAAAATAAATAACCAAAACTCAAAAAACTAACA (U) CAACGCTACTCTACGCTAAAAATAAATAACCGAAACTCGAAAAACTAACG (M)
**7**	64	1.90	0.006	-0.389	0.090	AAAAAACRTACCCCTCRACTATAAAATAAACATACCCRTATAATACCCCC
**8**	497	1.03	0.613	-0.097	0.684	ACAAACAAATACAAAAACCACTCAACCATAAAACTAAAATCAACACCACA (U) ACAAACAAATACGAAAACCGCTCAACCGTAAAACTAAAATCAACACCACG (M)
**9**	670	1.25	0.438	-0.161	0.497	AAACCAAAAAAACAAATCAATCCCAACCACAAAAAAAAAACCCCAAAACA (U) AAACCAAAAAAACGAATCAATCCCGACCGCAAAAAAAAAACCCCAAAACG (M)
**10**	706	1.26	0.262	0.022	0.928	RACCCAAAATCAAAAACRCCRCAAAAACAACAACTAAAAACCAAAAAAAC
**11**	925	1.94	0.008	-0.284	0.225	CCTTAAAATTACCCCTTAATTCCCTAACAACAAAAAACRAACCCTTATTC
**12**	937	1.25	0.311	-0.250	0.287	TCCCTACAAAACCCTTAAAATTACCCCTTAATTCCCTAACAACAAAAAAC
**13**	1057	1.67	0.032	-0.103	0.664	CCATTATTTAAATAAAAATTATATCTTAAATCAATACAACRACCTACCCC
**14**	**1119**	**1.50**	**0.041**	**-0.638**	**0.002**	ATTTCTAAACAACTAACTTTTTAAAATCCCATTTTCACCCTTCTATAACA (U) ATTTCTAAACGACTAACTTTTTAAAATCCCGTTTTCACCCTTCTATAACG (M)
**15**	**1158**	**2.06**	**0.028**	**-0.522**	**0.018**	RTAATTTTAAAAAACRACTATCAAACCATATTACCTATCCATTTCTAAAC

Site, distance to transcription start site; FC, fold change of beta value (cancer *vs*. paired normal tissue); Pearson's correlation, Pearson's correlation between DNA methylation level and *TRIM67* expression.

**Table 3:** Top 20 upregulated mRNAs identified by RNA sequencing (log_2_ fold change >1, *P*<0.05)

Gene Name	Gene Description	log_2_ Fold Change	*P*
EIF3C	eukaryotic translation initiation factor 3 subunit C	18.6087987	3.24E-20
SDAD1	SDA1 domain containing 1	17.8933647	4.94E-12
LDLR	low density lipoprotein receptor	17.8567578	1.04E-16
PMS2	PMS1 homolog 2, mismatch repair system component	17.5701522	9.69E-17
DDX24	DEAD-box helicase 24	17.4336615	3.53E-12
IGF2BP2	insulin like growth factor 2 mRNA binding protein 2	17.3794122	9.43E-08
MRPL48	mitochondrial ribosomal protein L48	17.3358904	6.70E-07
ARL4C	ADP ribosylation factor like GTPase 4C	16.8662486	5.24E-16
SMG7	SMG7, nonsense mediated mRNA decay factor	16.7889392	7.55E-15
AL133352.1	-	16.7885144	4.32E-12
IFI6	interferon alpha inducible protein 6	16.6868835	1.02E-09
SLC35A2	solute carrier family 35 member A2	16.5691634	9.07E-07
AC010531.1	-	16.3074143	2.91E-09
ALG8	ALG8, alpha-1,3-glucosyltransferase	16.3050405	1.52E-07
PRKACB	protein kinase cAMP-activated catalytic subunit beta	16.2828954	1.13E-14
DHPS	deoxyhypusine synthase	16.2554958	2.49E-05
TRAF7	TNF receptor associated factor 7	16.1516508	4.00E-09
DPP9	dipeptidyl peptidase 9	16.1141761	2.33E-11
ADGRG6	adhesion G protein-coupled receptor G6	16.075702	2.26E-12
MGA	MGA, MAX dimerization protein	15.9977382	6.83E-19

**Table 4:** Top 20 downregulated mRNAs identified by RNA sequencing (log_2_ fold change <-1, *P*<0.05)

Gene Name	Gene Description	log_2_ Fold Change	*P*
COPB2	coatomer protein complex subunit beta 2	-19.363245	1.84E-23
RTFDC1	replication termination factor 2 domain containing 1	-17.806038	6.95E-14
DDX24	DEAD-box helicase 24	-17.59781	5.52E-11
TPD52	tumor protein D52	-17.575332	4.96E-08
ANXA5	annexin A5	-16.9959	7.47E-08
RNF111	ring finger protein 111	-16.981478	2.91E-16
BCKDK	branched chain ketoacid dehydrogenase kinase	-16.919981	2.76E-09
MDH2	malate dehydrogenase 2	-16.81545	2.41E-11
SRPX	sushi repeat containing protein, X-linked	-16.702299	8.78E-08
MRPL28	mitochondrial ribosomal protein L28	-16.649013	1.42E-06
FBXO9	F-box protein 9	-16.629698	4.15E-10
ME2	malic enzyme 2	-16.584904	2.39E-11
MAPK11	mitogen-activated protein kinase 11	-16.550747	8.84E-12
GPAT2	glycerol-3-phosphate acyltransferase 2, mitochondrial	-16.52548	8.52E-12
BUD13	BUD13 homolog	-16.418643	1.33E-06
SARAF	store-operated calcium entry associated regulatory factor	-16.377437	6.96E-12
ARFGAP2	ADP ribosylation factor GTPase activating protein 2	-16.308007	2.99E-05
MOGS	mannosyl-oligosaccharide glucosidase	-16.287712	1.29E-05
CAMTA2	calmodulin binding transcription activator 2	-16.262853	1.72E-12
CCNB1IP1	cyclin B1 interacting protein 1	-16.248718	6.82E-10

## Abbreviations

CRC: colorectal cancer
TRIM67: tripartite motif-containing 67
TCGA: the cancer genome atlas
MAPK: mitogen-activated protein kinase
shRNA: short hairpin RNA
CCK-8: cell counting kit-8
